# Study of Texture Indicators Applied to Pavement Wear Analysis Based on 3D Image Technology

**DOI:** 10.3390/s22134955

**Published:** 2022-06-30

**Authors:** Yutao Li, Yuanhan Qin, Hui Wang, Shaodong Xu, Shenglin Li

**Affiliations:** 1 School of Civil Engineering, Chongqing University, Chongqing 400045, China; liyutao@cqu.edu.cn (Y.L.); qinyuanhan@cqu.edu.cn (Y.Q.); 2 Key Laboratory of New Technology for Construction of Cities in Mountain Area (Chongqing University), Ministry of Education, Chongqing 400045, China; 3 School of Microelectronics and Communication Engineering, Chongqing University, Chongqing 400045, China; xushaodong@cqu.edu.cn; 4 School of Software Engineering, Chongqing University of Posts and Telecommunications, Chongqing 400065, China; lisl@cqupt.edu.cn

**Keywords:** pavement texture, feature extraction, texture spectrum, gray level co-occurrence matrix, fractal theory, information entropy

## Abstract

Pavement texture characteristics can reflect early performance decay, skid resistance, and other information. However, most statistical texture indicators cannot express this difference. This study adopts 3D image camera equipment to collect texture data from laboratory asphalt mixture specimens and actual pavement. A pre-processing method was carried out, including data standardisation, slope correction, missing value and outlier processing, and envelope processing. Then the texture data were calculated based on texture separation, texture power spectrum, grey level co-occurrence matrix, and fractal theory to acquire six leading texture indicators and eight extended indicators. The Pearson correlation coefficient was used to analyse the correlation of different texture indicators. The distinction vector based on the information entropy is calculated to analyse the distinction of the indicators. High correlations between *ENE* (energy) and *ENT* (entropy), *ENT* and *D* (Minkowski dimension) were found. The *CON* (contrast) has low correlations with *HT* (macro-texture power spectrum area), *ENT* and *D*. However, the differentiation of *ENE* and *HT* is more prominent, and the differentiation of the *CON* is smaller. *ENE*, *ENT*, *CON* and *D* indicators based on macro-texture and the corresponding original texture have strong linear correlations. However, the microtexture indicators are not linearly correlated with the corresponding original texture indicators. *D*, *WT* (micro-texture power spectrum area) and *ENT* exhibit high degrees of numerical concentration for the same road sections and may be more statistically helpful in distinguishing the characteristics of the pavement performance decay of the road sections.

## 1. Introduction

Many scholars have tried to establish the relationships between pavement texture and pavement performance, such as surface ageing [1,2], skid resistance [1,3,4] and pavement noise generation [5,6]. These pieces of research show that the influence of pavement texture on pavement performance is very prominent. In addition, researchers believe texture features can be used in the design of pavement performance rather than just evaluation. Chu and Fwa [4] pointed out that different surface designs may be required for pavement skid resistance for different vehicle speeds and geometric alignments. Ktari et al. [7] found that the interface texture parameters significantly affect shear strength. Pratico and Astolfi [8] verified that macrotexture (MTD) and pendulum test values (PTV) could be explained and predicted based on simple physical and geometric models. Zou et al. [9] proved that pavement microtexture has a more significant effect on SFC than macrotexture. Fwa [10] demonstrated the conventional single-point representation of the skid resistance threshold and the two-parameter skid resistance models of Penn State and PIARC could not provide the needed information for wet weather driving risk assessment. Though evaluating asphalt concrete (AC) and gypsum asphalt (SMA) polished with real car tires, Xiao et al. [11] found the texture roughness (material area ratio and two-dimensional power spectral density) varied asynchronously at all observed scales (0.1 to 80 mm).

Above all, texture feathers may have more possibilities to reflect pavement performance decay. We need to find a way to evaluate texture indicators, not just correlated with anti-slip or noise. Spatial pattern analysis of the digital images (fractal dimension) distinguished between light, moderate and heavy degradation but failed to make a fine distinction between moderate levels of wear [12]. Chen et al. [13] developed a cost-effective and relatively accurate image-based texture analysis method (ITAM) based on digital image processing and spectral analysis techniques. Mean Texture Depth (MTD) calculated with an image-based procedure can be used instead of the MTD measured with the sand patch method, as the coefficient of determination is 0.99, and the standard error is 0.061 mm [14]. It was found that the parameter ω (the ratio between the volume of the pavement model and the current cutting depth) can be used in combination with traditional characterisation parameters to evaluate the macro-texture quality of pavements [15]. 3D-ITAM was validated as an effective method to characterise asphalt mixture surface macro- and microtexture properties by comparing the results from 3D-ITAM with those from SPM and HFT methods [16]. Medeiros Jr. et al. [17] further confirmed the accuracy of pavement macro-texture parameters from the digital image processing technique. Three-dimensional models constructed by stereo infrared scanning distinguished between all but the smoothest texture classes [12].

The development of 3D scanning and image technology has provided a more convenient way to obtain 3D texture data. Texture changes are more pronounced than SFC, MTD, and other test indicators. Extracting richer and more comprehensive texture feature information can help carry out road performance-related studies such as interface relationship, performance decay, and mix design of asphalt pavements based on the depth and multidimensional profiling of the apparent texture.

After data acquisition and indicators construction, we need to find a suitable method to evaluate the rationality of the indicators. Pearson correlation analysis has been used to find the relation between texture and other performance indicators [18] and exclude indicators with strong correlations among texture indicators to eliminate potential multicollinearity in model development [19]. We processed texture data by four methods: texture separation, texture power spectrum, grey level co-occurrence matrix, and fractal theory. The relevant feature indicators are compared and analysed to obtain less correlated, more distinguishable, and more suitable indicators for texture separation and wear study.

## 2. Data Acquisition

This study used the Gocator high-speed 3D laser contour sensor with a maximum field of view (FOV) of 365 mm and 1280 laser line contour points to collect texture data. In this test, the resolution in the x-direction was 0.171 mm, the resolution in the y-direction was 0.116 mm, the resolution in the z-direction was 0.013 mm, and the set motion speed in the y-direction was 50 mm/s.

**Figure 1:**
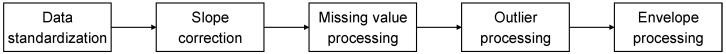
Pre-processing of texture data.

We selected three road sections with different wear levels for 50 m each and tested every 1 m. Furthermore, rutting plate specimens of AC-13 and SMA-13, the commonly used mix types for road surface layers, were prepared for texture indicator analysis. The pre-processing of texture data was completed by data standardisation (DS), slope correction (SC), missing value (MP) and outlier processing (OP), and envelope processing (EP), which is shown in Figure 1.

### 2.1. Data Standardisation (DS)

The samples need to be the same size to eliminate the influence of the data size on the conclusion. For the measured pavement planes, their sizes need to be unified as a picture with width multiplied by a size equal to 1123 × 1024 pixels and a texture information matrix with rows multiplied by columns equal to 1024 × 1123. The measured rutting plate and Marshall specimens were cut around to obtain an inner joint rectangle with width multiplied by 451 pixels × 359 pixels and a texture information matrix with rows multiplied by columns equal to 359 × 451.

### 2.2. Slope Correction (SC)

The texture data need to be corrected if the measured pavement has a certain slope concerning the horizontal surface. The road surface may have a horizontal and vertical slope, and the horizontal slope is generally between 1.0% and 2.0%. The maximum vertical slope does not exceed 8%, and this limit decreases as the design speed increases, so the vertical slope correction is generally performed. It is necessary to fit a plane to the texture data first, which can be achieved using the least-squares method or the more accurate Random Sample Consensus (RANSAC) algorithm.

The RANSAC algorithm randomly selects points for plane fitting, and the plane with the highest frequency of occurrence is the final fitted plane, so its accuracy is higher [20]. The RANSAC algorithm is used for the immediacy plane fitting for the texture data obtained from the above measurements. Then its plane equation can be obtained as follows.

(1) z=Ax+By+C

where *z*: information about the elevation of a point of the measured pavement, *x*, *y*: information about the plane position of a point of the measured pavement, *A*, *B*, *C*: constants, coefficients of the plane equation.

Since the maximum longitudinal slope does not exceed 8%, an approximate calibration can be made by processing only the elevation data to achieve the slope correction. That is, the calibrated elevation is:

(2) $$z_{i}^{\prime }=z_{i}-(Ax+By+C)$$

where $z_{i}$ is the elevation of a point before the calibration, and $z_{i}^{\prime }$ is its elevation after the calibration.

**Figure 2:**
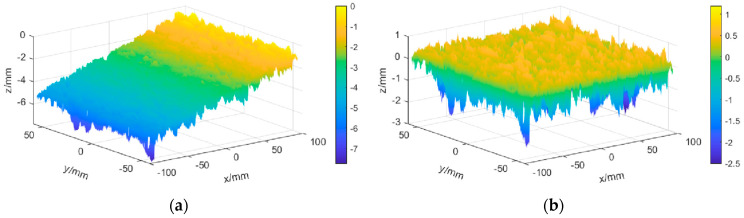
SC of pavement texture. (**a**) Pavement texture before SC. (**b**) Pavement texture after SC.

The pavement before and after slope correction can be obtained using MATLAB for programming calculation, as shown in Figure 2a,b.

### 2.3. Missing Value Processing (MP)

**Figure 3:**
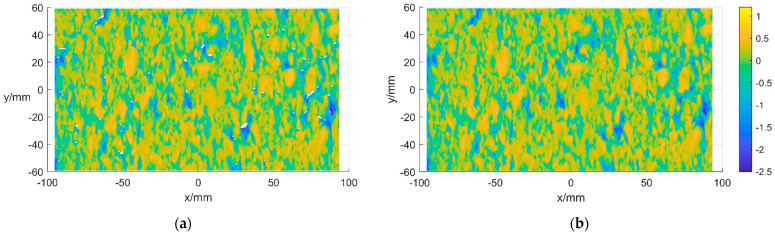
MP of pavement texture. (**a**) Pavement texture before MP. (**b**) Pavement texture after MP.

Some missing values may be generated for various reasons when measuring texture data. Suppose it is caused by the objective existence of gaps on the road surface and other reasons. The fixed value filling can be replaced with a value much lower than the lowest point of the texture data. If it is caused by the failure of the instrument or human operation error, the interpolation filling method can be used. In the case of more missing values at the edges, the adjacent rows and columns of data can be deleted to reduce the impact of large area interpolation on the overall texture analysis processing. MATLAB R2020a is used to detect the location of the missing values first, and then the third spline interpolation method is used to process them, and their local before-and-after comparisons are shown in Figure 3a,b.

The MP fills a small number of data gaps caused by the inability of the instrument to measure part of the seam due to the reflection of the laser, making the texture data more complete and the subsequent analysis more reasonable and accurate.

### 2.4. Outlier Processing (OP)

Outliers are one of the possible situations that can also occur in the measured texture data and can be divided into two types, pseudo-anomalies and true anomalies. The former can be judged in the field based on their measurement coordinates and reflect the actual condition of the pavement and usually do not need to be dealt with, while the latter is caused by instrument failure or human operation and need to be changed. Otherwise, the final results may be affected. Suppose the number of true anomalies is too high. In that case, it is necessary to consider whether the experiment has made an error to decide whether to discard the data and re-measure it.

Outliers can be judged by the median absolute deviation (MAD) algorithm, distance, clustering and density. Among them, the absolute median deviation algorithm is a commonly used method. For a data set $\left\{x_{1},x_{2},\cdots ,x_{n}\right\}$, first, calculate its median $x_{m}$, then calculate the total deviation of the data points from the median $d_{i}=\left|x_{i}-x_{m}\right|,i=1,2,\cdots ,n$, then calculate the median of the absolute deviation, $d_{m}$ and finally obtain the distance of all data points from the centre $l_{i}=\frac{d_{i}}{d_{m}}$. Usually, $d_{m}$ can be used as a consistent estimate of the standard deviation σ. The relationship between the two is $\sigma =k\cdot d_{m}$, where *k* is a constant factor with different values depending on the data distribution, k is usually equal to 1.4286 for normal distribution. The mean value μ is calculated, and then the values of the $\mu +3\sigma \cdot k\cdot d_{m}$ and $\mu -3\sigma \cdot k\cdot d_{m}$ are calculated to determine the range of statistically significant standard data points. Data points outside the interval $\left[\mu -3\sigma \cdot k\cdot d_{m},\mu +3\sigma \cdot k\cdot d_{m}\right]$ are considered outliers [21].

**Figure 4:**
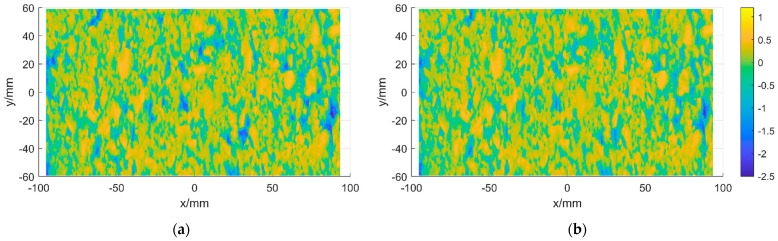
OP of pavement texture. (**a**) Pavement texture before OP. (**b**) Pavement texture after OP.

The absolute median difference algorithm is a distance value method that is robust against outlier data and amplifies the effect of outliers. A method similar to that used to fill in missing values can be used when correcting outliers. The MAD algorithm is used to determine its position for the data here, and the interpolation method is used to process its local outliers. Its local OP results before and after comparison are shown in Figure 4a,b.

### 2.5. Envelope Processing (EP)

**Figure 5:**
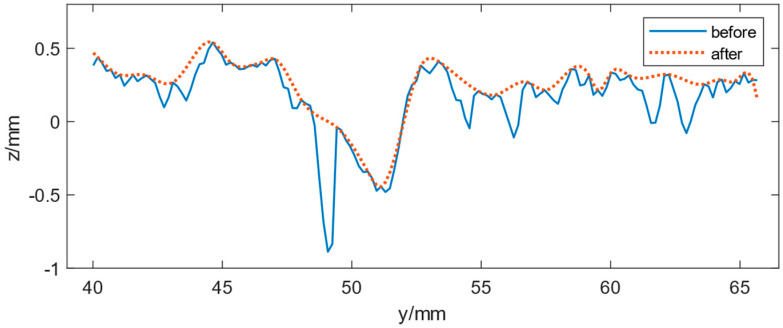
EP of pavement texture (partial).

The tire is more likely to contact the protruding part of the pavement, while the depressed part does not. Therefore, the envelope of texture data is needed to better calculate the real contact surface between the tire and the road surface to establish a connection with the road performance indicators. There are three commonly used methods for envelope calculation. The first one is to filter the texture data with a certain length of Hilbert FIR filter after Discrete Fourier Transform (DFT) to obtain the envelope. The second one is to return the root-mean-square envelope by a sliding window of a certain length. The third one is to find the wave peaks, then take out some peaks separated by a certain number of waves and obtain the envelope by applying a spline interpolation method. A suitable envelope is obtained by changing some parameters in these algorithms and then cross-validating them with the pavement evaluation parameters [22]. For the data here, each wave crest was collected and then interpolated with the spline interpolation method and combined with the pavement evaluation parameters to obtain the envelope. The result of the profile’s envelope based on partial data is shown in Figure 5.

The envelope-processed pavement texture section better characterises the tire-pavement contact, making the subsequent analysis more accurate and meaningful for wear analysis.

## 3. Methodology

### 3.1. Texture Indicators Calculation Methods

#### 3.1.1. Texture Separation

By considering the distance between the texture profile and the reference surface as a smooth random function, it is possible to define the distance between two recurring constructions as the wavelength of the texture. Different texture scales can affect pavement performance indicators differently, so it is necessary to classify the textures. Pavement textures are usually divided into three categories by wavelength, including micro-texture (0~0.5 mm), macro-texture (0.5~50 mm), and mega-texture (50~500 mm) [6]. The Fourier analysis method can decompose the texture into sinusoidal components of different wavelengths and amplitudes, which occupy different proportions of the total texture data. Therefore, the texture data indicators of different wavelengths can be calculated to reflect the effects of different wavelength textures and their distribution characteristics in the total texture data.

**Figure 6:**
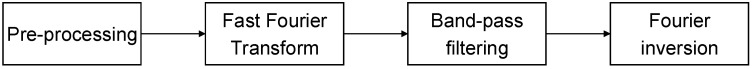
Flow chart of texture separation.

For a set of pavement texture data, each column (i.e., its y-direction data) is subjected to a Fast Fourier Transform (FFT), which transforms it from the spatial domain to the frequency domain with frequency units of mm^−1^. Then a bandpass filter is designed to filter the texture data. The wavelength of micro-texture is 0~0.5 mm, and the wavelength of 0.5 mm corresponds to a frequency of 2 mm^−1^. While the resolution in the z-direction at the measurement time is 0.013 mm, the maximum frequency after FFT corresponding to this size is 38.46 mm^−1^. Therefore, a bandpass filter of 2~38.46 mm^−1^ can be designed to filter out the micro-texture. The wavelength of macro-texture is 0.5~50 mm, and the frequency corresponding to the 50 mm wavelength is 0.02 mm^−^^1^, so a bandpass filter of 0.02~2 mm^−1^ could be used for the macro-texture. However, since the components with frequencies below 0.02 mm^−1^ are small and negligible, the lower cutoff frequency of 0.02 mm^−1^ is difficult to achieve in bandpass filters from 0.02 to 2 mm^−1^. A low-pass filter with a frequency of 2 mm^−1^ is used to approximate the bandpass filter of 0.02~2 mm^−1^, which is more effective and avoids large distortion. In addition, according to Nyquist’s sampling theorem, the selected sampling rate should be greater than twice the highest frequency of the original signal, which can avoid the occurrence of spectral aliasing and thus restore the original signal without distortion. Finally, the Fourier inversion can reduce the frequency domain’s texture data to the spatial domain’s texture data. The computational flow chart is shown in Figure 6.

#### 3.1.2. Texture Power Spectrum Density

The power spectral density can be used to assess the level of macro-texture and micro-texture of pavement. First, the macro-texture and micro-texture power spectra are calculated separately using the PWelch function in Matlab. Then, the PWelch function uses the input signal power spectral density (PSD) estimates found by Welch’s overlapping segment averaging estimator. For the pavement, the window function is specified as the Hamming window, and the length of the Hamming window is 1024. The number of overlapping samples, defined as a positive integer less than the length of the window, is taken here as 512. The number of points of DFT is 256, and the length of the segments is 2048. The sampling rate is defined as the number of samples per unit, taken here as 100 mm^−1^. For the specimens, the Hamming window length is 256, the number of overlapping samples is 128, the number of DFT points is 512, and the sampling rate is 100 mm^−1^. The area under the power spectral density curve for the frequencies in its range is used as the characteristic indicators of macro-texture and micro-texture for this pavement section (i.e., this column of data in the Y-direction). Finally, the data of all road sections are averaged to obtain this pavement’s power spectral density indicators. This method avoids the influence of the difference in the number of texture data columns on the conclusion.

#### 3.1.3. Grey Level Co-Occurrence Matrix

Grey level co-occurrence matrix (GLCM) is a processing method for texture data used in texture feature representation in high-precision manufacturing. It represents the joint probability density distribution of the simultaneous occurrence of two pixels separated by a certain distance in the whole image according to a certain translation direction. The texture features can be extracted by analysing the spatial relationship of the grey values of pixels separated by a certain distance in space and rewriting them with texture descriptors [23,24].

For the texture image M×M, take any point $A\left(x,y\right)$, transform its horizontal and vertical coordinates to obtain $A^{\prime }\left(x+p,y+q\right)$, and set the grey value of the point pair $A,A^{\prime }$ as $\left(g_{1},g_{2}\right)$. Different grayscale values $\left(g_{1},g_{2}\right)$ can be obtained for different points. If the number of grayscale levels is G, then there are several possible grayscale values $G^{2}$. The occurrences are counted, normalised to frequency, and arranged as a square matrix to obtain the grayscale co-occurrence matrix P for each grayscale value. The coordinate transformation value $\left(p,q\right)$ needs to be chosen according to the texture characteristics. A smaller value $\left(1,0\right)$ representing a horizontal pixel pair, i.e., a 0° scan, is chosen for finer textures. It is also reasonable to choose other values such as $\left(1,1\right),\left(0,1\right),\left(-1,-1\right)$, whose scanning angle will also change to 45°, 90°, 135°, etc. Haralick [25] proposed 14 statistics such as energy, entropy, contrast, uniformity, correlation, etc. Contrast, entropy, and energy can be chosen to describe the texture features. These indicators can be computed based on the original or separated texture.

Energy represents the thickness and uniformity of texture distribution, and its calculation method is shown in (3).

(3) $$ENE=\sum_{i,j}P{\left(i,j\right)}^{2}$$

Entropy reflects the complexity of the distribution, and its calculation method is shown in (4).

(4) $$ENT=-\sum_{i,j}P\left(i,j\right)\cdot \ln P\left(i,j\right)$$

The contrast reflects the sharpness of the image and the groove depth of the texture. Its calculation method is shown in (5).

(5) $$CON=\sum_{i,j}{\left(i-j\right)}^{2}\cdot P(i,j)$$

where $P\left(i,j\right)$ is the element of the *i*_th_ row and *j*_th_ column of the grayscale co-generation matrix.

#### 3.1.4. Fractal Theory

Mandelbrot has defined fractals twice [26,27]. In 1986, Mandelbrot defined a fractal as a shape composed of parts similar to the whole. This definition emphasises the self-similarity of fractal sets [28]. Fractal theory studies its properties and applications. It has two principles, the principle of self-similarity and iterative generation, characterising fractals under the usual geometric transformations independent of scale. Self-similar shapes can be identical or similar statistically, which implies recursion. The value of a graph with no fractal structure is exactly the spatial dimension of this structure, but it may not be an integer for a fractal pattern with infinite details [29,30].

The texture of a certain section of a road surface also has self-similarity, and the fractal dimension can be used to evaluate the texture condition of the road surface. There are usually two types of fractal dimensions, the Hausdorff dimension and the Minkowski dimension, the latter also known as the box-counting dimension. The Hausdorff dimension is the most commonly used dimension, and its expression is shown in (6).

(6) $$K=L^{D_{f}}$$

The equation is equivalent to (7).

(7) $$K={\left(\frac{1}{L}\right)}^{-D_{f}}$$

Taking the natural logarithm and sorting gives (8).

(8) $$D_{f}=\frac{\ln K}{\ln L}$$

where L is the number of times a certain shape expands along each independent direction, and K is the number of times the resulting fractal occupies a range of space relative to the original shape.

The idea of the Minkowski dimension is to cover the set using disjoint boxes. Firstly, each point’s height is represented by a grey value with a grey level G. Secondly, the set is divided into several rectangles with the size of $R\times R\times R^{\prime }$, where $R^{\prime }=R\cdot \frac{G}{M}$, ensuring the number of rectangles in each direction is equal in the 3D coordinates. In the position (i,j) of the grid R×R, find the maximum grey value u, the minimum grey value, and then the minimum number of squares covering the minimum to the maximum grey value is (9).

(9) $$n_{R}(i,j)=u-b+1$$

Then summing over the entire grid of R×R is (10).

(10) $$N_{R}=\sum_{i,j}n_{R}(i,j)$$

The fractal dimension of the whole texture image is shown in (11) [31]. Similar to the previous indicators, this indicator can be calculated based on the original and separated textures.

(11) $$D=\underset{R\to \infty }{\lim}\frac{\log(N_{R})}{\log\left(\frac{1}{R}\right)}$$

### 3.2. Indicators Evaluation Method

Reasonable indicators should have weak linear correlations to eliminate the effect of subsequent model building of multicollinearity, which the Pearson correlation coefficient matrix can measure. Furthermore, they need to have a significant degree of distinction to amplify the differences between indicators of different textures, which can be calculated by distinction vectors based on information entropy.

The data sets are first dimensionless to obtain the dimensionless matrix of the texture indicator set $X=\left(x_{ij}\right)n\times m$ (*n* is the set number, and *m* is the indicator number). The Pearson correlation coefficients of each two indicators are calculated by (12).

(12) $$s_{pq}=\frac{\mathrm{cov}\left(p,q\right)}{\sigma_{p}\sigma_{q}}$$

where $s_{pq}$ is the element of the correlation matrix ***S***. The meanings of *p* and *q* are the *p*_th_ and the *q*_th_ columns of the dimensionless matrix ***X***. The cov(p,q) is the covariance between *p* and *q*, and $\sigma_{p},\sigma_{q}$ are the standard deviations of *p* and *q*.

Next, the entropy of the *i*_th_ texture indicator is calculated by (13).

(13) $$H_{i}=-k\sum_{j=1}^{n}r_{ij}\ln r_{ij},i=1,2,\cdots ,m$$

where $r_{ij}=\frac{x_{ij}}{\sum_{j=1}^{n}x_{ij}}$, $k=\frac{1}{\ln n}$.

Then the differentiation of the *i*_th_ texture indicator is

(14) $$g_{i}=1-\frac{H_{i}}{\sum_{j=1}^{m}H_{j}},i=1,2,\cdots ,m$$

where $g_{i}$ is the element of the distinction vector ***G***.

## 4. Results and Discussion

### 4.1. Texture Indicators Calculation Results

#### 4.1.1. Texture Separation

**Figure 7:**
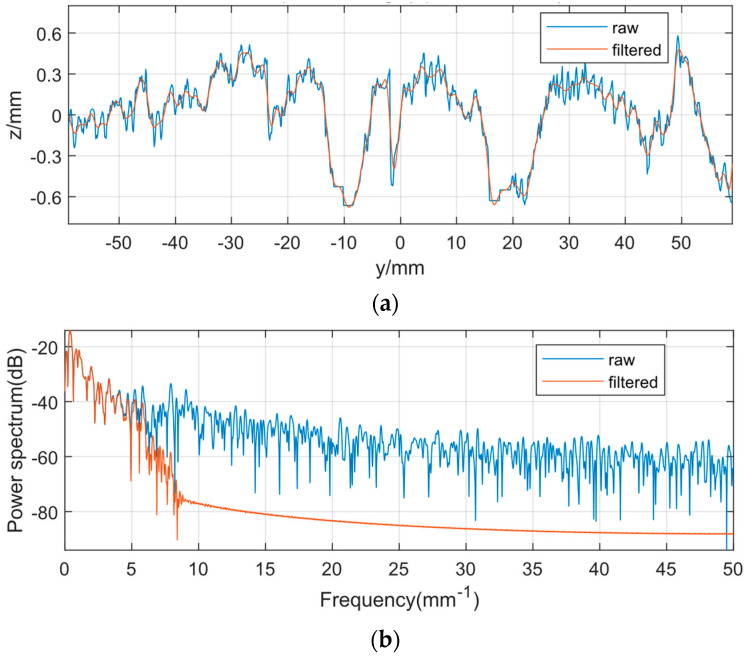
Macro-texture filtering results. (**a**) Low-pass filtering of texture (Fpass = 2 mm^−1^). (**b**) Power spectrum of low-pass filtered texture.

**Figure 8:**
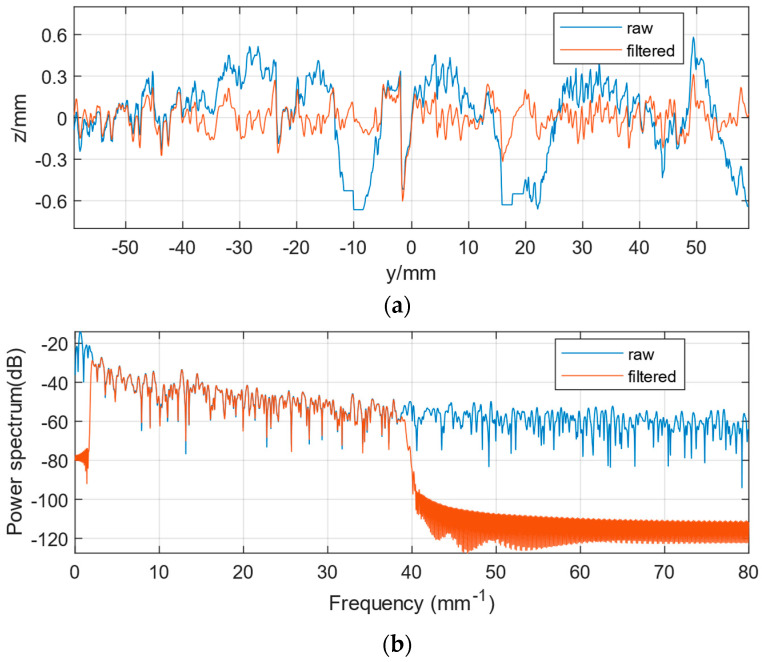
Micro-texture filtering results. (**a**) Band-pass filtering of texture (Fpass = [2 38.46] mm^−1^). (**b**) Power spectrum of bandpass filtered texture.

The macro-texture and micro-texture filtering results of one of the pavement sections are shown in Figure 7 and Figure 8.

As seen in Figure 7a, the macro-texture is obtained by filtering the texture using a low-pass filter with a pass frequency of 2 mm^−1^, which shows the overall undulating trend of the texture and ignores its subtle local variations. The overlapping parts of the power spectrum curves before and after filtering are shown in Figure 7b. Similarly, as seen in Figure 8a, the micro-texture is obtained by filtering the texture using a bandpass filter passing through the frequency interval from 2 to 38.46 mm^−1^, exhibiting subtle local variations and ignoring the overall undulating trend of its texture. The overlapping parts of the power spectrum curves before and after filtering are shown in Figure 8b.

**Figure 9:**
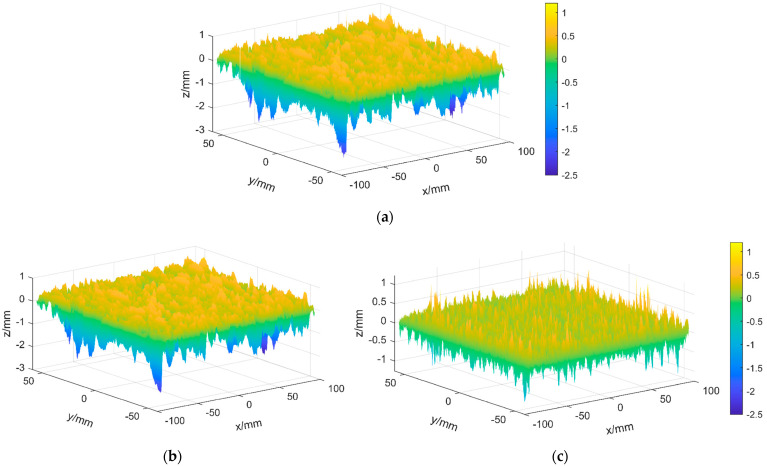
Comparison before and after texture separation. (**a**) Original texture. (**b**) Macro-texture. (**c**) Micro-texture.

Figure 7 and Figure 8 show that the filter and sample rate settings do well in separating the macro-texture and micro-texture portions of the pavement texture. The 3D comparison figures before and after texture separation are shown in Figure 9, which demonstrate the effect of texture separation.

As can be seen in Figure 9b, the macro-texture after texture separation characterises the overall undulating trend of the original texture. In Figure 9c, the micro-texture shows only the fine features of the texture. Therefore, the original texture in Figure 9a is well separated.

#### 4.1.2. Texture Power Spectrum Density

**Figure 10:**
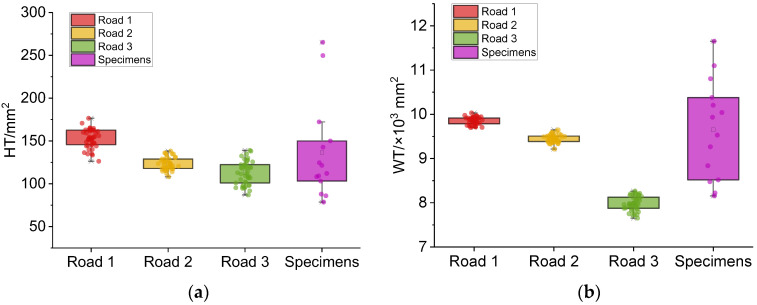
Areas under the PSD curve for macro-texture and micro-texture of different roads and specimens. (**a**) *HT*s of different roads and specimens. (**b**) *WT*s of different roads and specimens.

The area distributions under the power spectral density curve of macro-texture and micro-texture for different roads and specimens are shown in Figure 10.

As shown in Figure 10, the indicators’ distribution of roads 1 to 3 is more concentrated, while the same indicators’ distribution of specimens is more discrete. For road sections, the *HTs* are more discrete than the *WTs*. In contrast, the specimens’ *WTs* demonstrate higher discreteness than the *HTs*.

It seems that the micro-texture of the same road section tends to be consistent, which is more representative of the pavement wear state of the road section. The specimens’ *HTs* may have certain representativeness, but the representativeness of specimens’ *WTs* is insufficient.

#### 4.1.3. Grey Level Co-Occurrence Matrix (GLCM)

**Figure 11:**
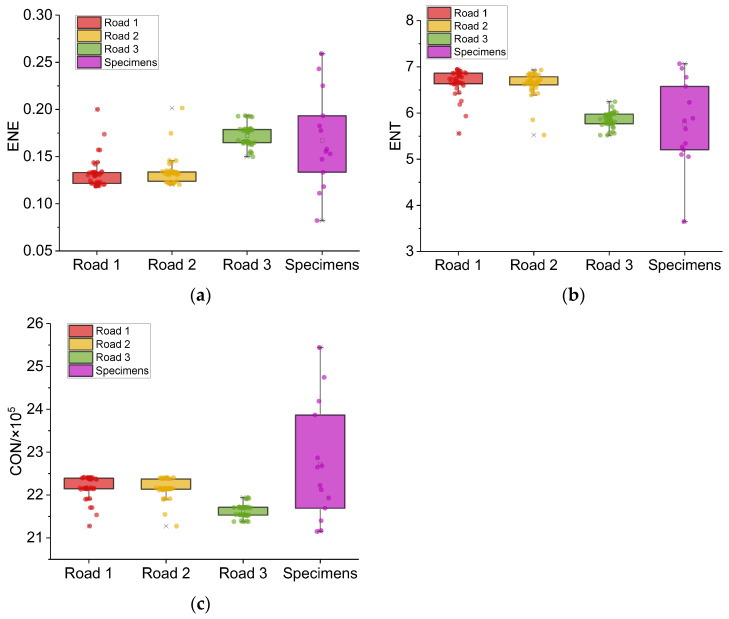
GLCM indicators of different roads and specimens. (**a**) *ENEs* of different roads and specimens. (**b**) *ENTs* of different roads and specimens. (**c**) *CONs* of different roads and specimens.

The distributions of the grey level co-occurrence matrix indicators for texture images of different pavement and specimen images are shown in Figure 11.

Similar to the texture spectrum indicator, the GLCM indicators of roads 1 to 3 show similar concentration trends, while the specimens show dispersion. Among the three indicators, *ENT* seems to perform best.

#### 4.1.4. Fractal Theory

**Figure 12:**
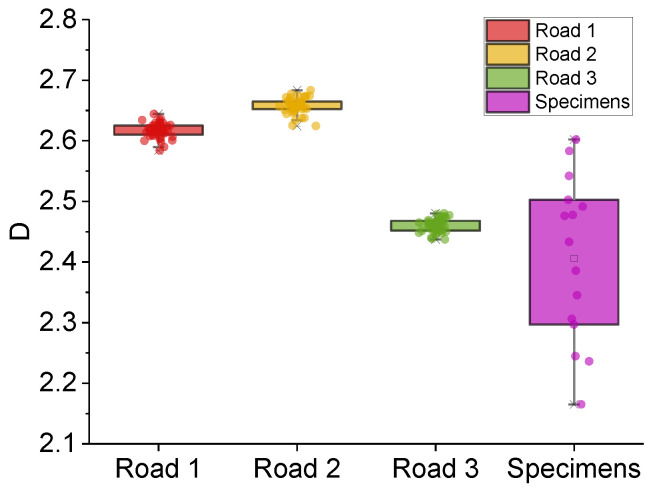
*D*s of different roads and specimens.

The Minkowski dimension *D* for texture images of roads 1 to 3 and specimens were distributed in the range of 2.1 to 2.7, as shown in Figure 12.

Figure 12 implies that *D* exhibits a high degree of numerical concentration for the same road section, which may be more statistically helpful in distinguishing the characteristics of the pavement performance decay. *D* can reflect the wear on different scales simultaneously, which is more representative. In addition, *D*s have similar distribution characteristics to the previous indicators, indicating that texture indicators’ general trends are consistent.

### 4.2. Comparative Analysis and Evaluation of Texture Indicators

#### 4.2.1. Pearson Correlation Coefficients Matrix

**Figure 13:**
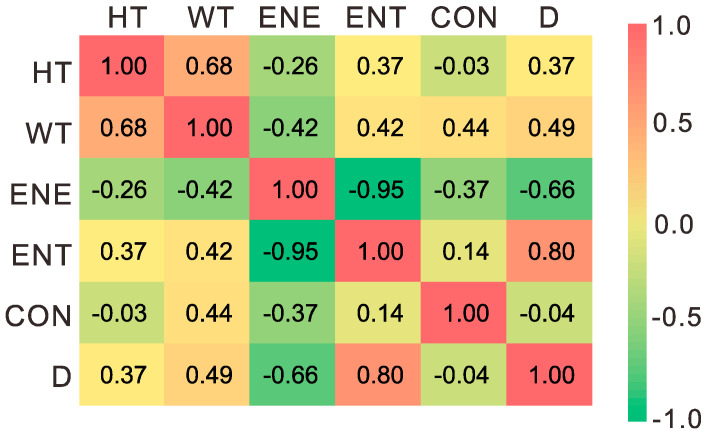
Pearson correlation coefficients matrix of different texture indicators.

Pearson correlation coefficients of the six indicators (*HT*, *WT*, *ENE*, *ENT*, *CON*, and *D*) were calculated, and the results are plotted in Figure 13.

As seen in Figure 13, *ENE* and *ENT* have a strong negative correlation, while *D* and *ENT* have a strong positive correlation. The texture information reflected by the two GLCM indicators (*ENE* and *ENT*) and the fractal indicator (*D*) has certain consistency, which means that these indicators have certain substitutability for each other. There are no obvious linear correlations between *CON* and other indicators, implicating that *CON* may be suitably used together with other indicators for multidimensional texture analysis.

#### 4.2.2. Distinction Vector

According to the information entropy theory, the differentiation degrees of *HT*, *WT*, *ENE*, *CON*, *ENT*, and *D* are calculated to obtain the distinction vector ***G*****_1_**. The result is shown in (15).

(15) $$G_{1}=\left(0.420\;0.100\;0.369\;0.084\;0.007\;0.020\right)$$

The distinction vector ***G*****_1_** indicates that the two indicators with the largest differentiation are *HT* and *ENE*, whose differentiation is more than twice as large as the differentiation of the other indicators. *HT* and *ENE* better reflect the gap between different textures and may be more suitable as texture indicators for wear analysis. The indicator with the smallest differentiation is *CON*, which may be not suitable for evaluating pavement wear, as the values of the specific indicators evaluated will be closer. *CON* represents some unique texture statistical information of pavement texture (Section 4.2.1), which may be used together with other texture indicators to study other performances (such as noise, anti-sliding, etc.) related to pavement texture features.

#### 4.2.3. The Effect of the Texture Separation on These Indicators

**Figure 14:**
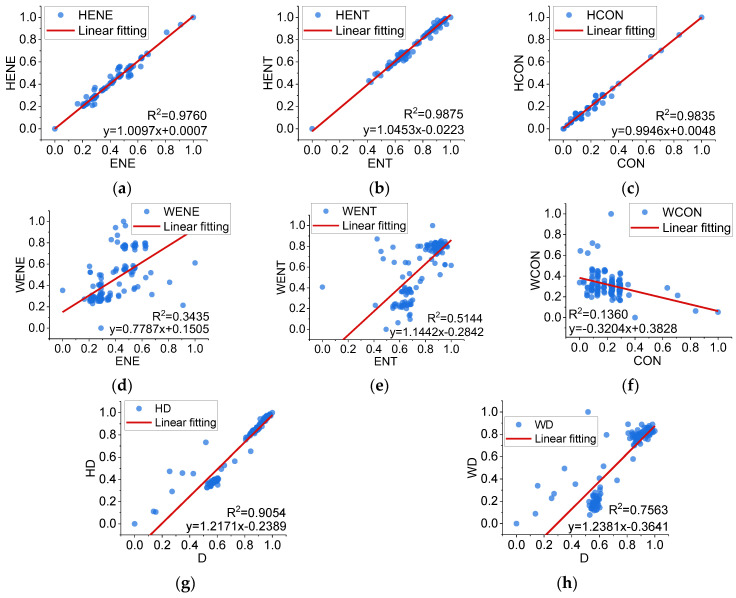
Linear fitting between the texture indicators before and after texture separation. (**a**) *ENE* and *HENE*. (**b**) *ENT* and *HENT*. (**c**) *CON* and *HCON*. (**d**) *ENE* and *HENE*. (**e**) *ENT* and *HENT*. (**f**) *CON* and *HCON*. (**g**) *D* and *HD*. (**h**) *D* and *WD*.

The grey level co-occurrence matrix and fractal theory can also be used to calculate indicators based on the filtered macro-texture and micro-texture data. The indicators before and after separation are linearly fitted, as shown in Figure 14.

As seen in Figure 14, the macro-texture indicators *HENE*, *HENT*, *HCON*, and *HD* have solid linear correlations with the original texture indicators *ENE*, *ENT*, *CON*, and *D*, and the *R*^2^ values of the linear fits are above 0.9. Except for *WD*, the micro-texture indicators are not linearly correlated with the original texture indicators. The macro-texture indicators seem to overlap more with the meanings expressed by the original texture indicators. The distinction vectors are calculated to analyse the effect of the texture separation on these indicators, as shown in (16).

(16) $$\begin{array}{l} G_{2}=\left(0.465\;0.463\;0.072\right)\\ G_{3}=\left(0.419\;0.427\;0.154\right)\\ G_{4}=\left(0.507\;0.491\;0.002\right)\\ G_{5}=\left(0.312\;0.507\;0.181\right) \end{array}$$

***G*_2_**~***G*****_5_** denote the differentiation vectors of *ENE*, *ENT*, *CON* and *D* indicators based on original, macro-texture and micro-texture, respectively. The differentiation degrees of the micro-texture indicators are much smaller than those of the original and macro-texture. For micro-texture indicators, the *ENT* and *D* differentiation degrees are more significant than those of *ENE* and *CON*.

The differentiation degrees of the macro-texture are similar to those of the original textures in ***G*_2_**~***G*_4_** but significantly higher in ***G*_5_**. As *D* has a greater differentiation than other indicators on the micro-scale, and its macro-texture has a higher differentiation than the overall one, its performance in wear characteristics seems to be more prominent. Although not significant enough, *ENT* also shows the same trend characteristics as *D*. It is more reasonable and effective to perform texture separation for *ENT* and *D* applied to the wear analysis.

## 5. Conclusions

We scanned texture data from 3 road sections and 14 rutting plate specimens of the AC-13 mix. Based on the texture data achieved, six statistical indicators were calculated. The Pearson correlation coefficient was used to analyse the correlation of different texture indicators. The distinction vectors based on the information entropy were calculated to analyse the distinction of the indicators. The effect of texture separation on calculating the grey level co-occurrence matrix and fractal indicators was investigated. The following conclusions were obtained:

1. *D*, *WT* and *ENT* exhibit high degrees of numerical concentration for the same road sections. The three indicators may be more statistically helpful in distinguishing the characteristics of pavement performance decay. *D* reflects different information from all the other statistical variables. The specimens do not possess all the actual pavements’ statistical characteristics.
2. *ENE*, *ENT* and *D* have a certain consistency, which means that these indicators have certain substitutability for each other. *CON* represents some unique texture statistical information.
3. *ENT* and *D* have greater differentiation than other indicators on the micro-scale, and its macro-texture has a higher differentiation than the overall one. Their performance in wear characteristics seems to be more prominent.
4. With high degrees of numerical concentration for the same road sections and differentiation degrees, *D*, PSD indicators, and *ENT* should be further studied based on more road sections or abraded specimens.

The fractal and texture-based energy indicators at different scales can provide better technical means for evaluating pavement wear and further be used to assess noise and skid resistance by multi-scale texture analysis. However, the practical engineering level comparison can hardly be carried out due to the coarse means of existing noise and skid resistance testing.

Further study can be carried out in conjunction with microscopic simulation modelling of noise or tire skid resistance performance to construct metrics suitable for early wear performance decay analysis of pavements.

## Data Availability

The data presented in this study are available on request from the corresponding author. The data are not publicly available due to the confidentiality requirements for unfinished research projects.

## References

[1] Kokkalis A.G., Tsohos G.H., Panagouli O.K. (2002). Consideration of fractals potential in pavement skid resistance evaluation. J. Transp. Eng..

[2] Flintsch G.W., McGhee K.K., Izeppi E.D.L., Najafi S. (2012). The Little Book of Tire Pavement Friction. Pavement Surf. Prop. Consort..

[3] Ueckermann A., Wang D., Oeser M., Steinauer B. (2015). Calculation of skid resistance from texture measurements. J. Traffic Transp. Eng. Engl. Ed..

[4] Chu L., Fwa T.F. (2016). Incorporating Pavement Skid Resistance and Hydroplaning Risk Considerations in Asphalt Mix Design. J. Transp. Eng..

[5] Anfosso-Lédée F., Do M.-T. (2002). Geometric Descriptors of Road Surface Texture in Relation to Tire-Road Noise. Transp. Res. Rec. J. Transp. Res. Board.

[6] Smit A., Trevino M., Garcia N.Z., Buddhavarapu P., Prozzi J. (2016). Selection and Design of Quiet Pavement Surfaces (No. FHWA/TX-16/0-6819-1). https://library.ctr.utexas.edu/ctr-publications/0-6819-1.pdf.

[7] Ktari R., Fouchal F., Millien A., Petit C. (2017). Surface roughness: A key parameter in pavement interface design. Eur. J. Environ. Civ. Eng..

[8] Pratico F.G., Astolfi A. (2017). A new and simplified approach to assess the pavement surface micro-and macrotexture. Constr. Build. Mater..

[9] Zou Y., Yang G., Cao M. (2021). Neural network-based prediction of sideway force coefficient for asphalt pavement using high-resolution 3D texture data. Int. J. Pavement Eng..

[10] Fwa T.F. (2017). Skid resistance determination for pavement management and wet-weather road safety. Int. J. Transp. Sci. Technol..

[11] Xiao S., Tan T., Xing C., Tan Y. (2020). A contribution to texture analysis of pavements under simulated polishing: Some new findings. Int. J. Pavement Eng..

[12] Matlack G.R., Horn A., Aldo A., Walubita L.F., Naik B., Khoury I. (2021). Measuring surface texture of in-service asphalt pavement: Evaluation of two proposed hand-portable methods. Road Mater. Pavement Des..

[13] Chen D., Sefidmazgi N.R., Bahia H. (2015). Exploring the feasibility of evaluating asphalt pavement surface macro-texture using image-based texture analysis method. Road Mater. Pavement Des..

[14] Puzzo L., Loprencipe G., Tozzo C., D’Andrea A. (2017). Three-dimensional survey method of pavement texture using photographic equipment. Measurement.

[15] Xin Q., Qian Z., Miao Y., Meng L., Wang L. (2017). Three-dimensional characterisation of asphalt pavement macrotexture using laser scanner and micro element. Road Mater. Pavement Des..

[16] Chen D. (2020). Evaluating asphalt pavement surface texture using 3D digital imaging. Int. J. Pavement Eng..

[17] Medeiros M., Babadopulos L., Maia R., Castelo Branco V. (2021). 3D pavement macrotexture parameters from close range photogrammetry. Int. J. Pavement Eng..

[18] Wei D., Li B., Zhang Z., Han F., Zhang X., Zhang M., Li L., Wang Q. (2018). Influence of Surface Texture Characteristics on the Noise in Grooving Concrete Pavement. Appl. Sci..

[19] Li Q.J., Zhan Y., Yang G., Wang K.C.P. (2020). Pavement skid resistance as a function of pavement surface and aggregate texture properties. Int. J. Pavement Eng..

[20] Nurunnabi A., Belton D., West G. (2014). Robust statistical approaches for local planar surface fitting in 3D laser scanning data. Isprs J. Photogramm. Remote Sens..

[21] Leys C., Ley C., Klein O., Bernard P., Licata L. (2013). Detecting outliers: Do not use standard deviation around the mean, use absolute deviation around the median. J. Exp. Soc. Psychol..

[22] Mathworks Inc. (2022). Signal Processing Toolbox User’s Guide.

[23] Unser M. (1986). Sum and Difference Histograms for Texture Classification. IEEE Trans. Pattern Anal. Mach. Intell..

[24] Marceau D.J., Howarth P.J., Dubois J.M., Gratton D.J. (1990). Evaluation Of The Grey-level Co-occurrence Matrix Method For Land-cover Classification Using Spot Imagery. IEEE Trans. Geosci. Remote Sens..

[25] Haralick R.M., Shanmugam K., Dinstein I. (1973). Textural Features for Image Classification. IEEE Trans. Syst. Man Cybern..

[26] Mandelbrot B.B. (1982). The Fractal Geometry of Nature.

[27] Feder J. (1988). Fractals.

[28] Lam A.D.K.T., Li Q. Fractal analysis and multifractal spectra for the images. Proceedings of the 2010 International Symposium on Computer, Communication, Control and Automation (3CA).

[29] Cai J., Zhou G. Study on Fractal Theory in Identifing Soil Fabric in Civil Engineering. Proceedings of the 2011 Fourth International Workshop on Chaos-Fractals Theories and Applications (IWCFTA).

[30] Li W., Zhao H., Guo J., Wang L., Yu J. A multi-scale fractal dimension based onboard ship saliency detection algorithm. Proceedings of the 2016 IEEE 13th International Conference on Signal Processing (ICSP).

[31] Sarkar N., Chaudhuri B.B. (1994). An efficient differential box-counting approach to compute fractal dimension of image. IEEE Trans. Syst. Man Cybern..

